# *S cerevisiae* genome as a confined equilibrium polymer brush

**DOI:** 10.1186/1756-8935-6-S1-P129

**Published:** 2013-04-08

**Authors:** Anton Goloborodko, Jon Matthew Belton, Geoffrey Fudenberg, Maxim Imakaev, Job Dekker, Leonid Mirny

**Affiliations:** 1 Department of Physics, Massachusetts Institute of Technology, Cambridge, 02143, MA, USA; 2 Department of Biochemistry and Molecular Pharmacology, University of Massachusetts, Worcester, 01605, MA, USA; 3 Graduate Program in Biophysics, Harvard University, Cambridge, 02115, MA, USA; 4 Division of Health Science and Technology, Harvard-MIT, Cambridge, MA, 02139, USA

## Introduction

A series of recent studies using optical and 3C-based experimental approaches [1,2] have shown that at a global level the yeast genome assumes a Rabl-like conformation with the chromosomal centromeres tethered to the spindle pole body and telomeres anchored on the nuclear periphery. This is strikingly different from human genomic organization, where chromosomes have been shown to assume fractal globule conformations with domains of active and inactive chromatin [3].

In this work, we study Rabl-like chromosomal organization using a computationally efficient polymer lattice model.

## Results

We find that our lattice polymer model predicts experimentally observed Hi-C contact maps with high precision (r~0.87). This demonstrates that a minimal equilibrium model can reconstitute the majority of the observed interaction patterns. Our model predicts that loci preferentially localize to different regions of the nucleus in 3D, depending on their genomic positions. In turn, this leads to different contact preferences between loci. This effect is most clearly demonstrated by the cross-like patterns of interactions between peri-centromeric regions, as observed in yeast Hi-C data. Centromere colocalization and excluded volume interactions cause chromatin fibers to extend away from the spindle pole body; this can be understood as a ‘polymer brush’ effect. In turn, the spatial localization of peri-centromeric loci becomes mainly determined by their genomic distances to the centromere; each locus preferentially forms contacts with other loci at similar distances from their respective centromeres. This dependence of spatial localization on genomic coordinates produces prominent cross-like patterns, as observed in *S. cerevisiae* Hi-C maps. We estimate this polymer brush effect to alter contact preferences at distances up to ~200 kbp from each centromere. Finally, our lattice model allow us to quickly predict the relative impact of fiber width, flexibility and linear compaction on chromosomal conformation. We find that the observed Hi-C maps are consistent with a range of fiber parameters.
